# *Neisseria meningitidis* Differentially Controls Host Cell Motility through PilC1 and PilC2 Components of Type IV Pili

**DOI:** 10.1371/journal.pone.0006834

**Published:** 2009-08-31

**Authors:** Philippe C. Morand, Marek Drab, Krishnaraj Rajalingam, Xavier Nassif, Thomas F. Meyer

**Affiliations:** 1 Department of Molecular Biology, Max-Planck-Institute for Infection Biology, Berlin, Germany; 2 Faculté de Médecine, Université Paris Descartes, Paris, France; 3 INSERM (Institut National de la Santé et de la Recherche Médicale) U567, Institut Cochin, Paris, France; 4 INSERM (Institut National de la Santé et de la Recherche Médicale) U570, Paris, France; University of British Columbia, Canada

## Abstract

*Neisseria meningitidis* is a strictly human pathogen that has two facets since asymptomatic carriage can unpredictably turn into fulminant forms of infection. Meningococcal pathogenesis relies on the ability of the bacteria to break host epithelial or endothelial cellular barriers. Highly restrictive, yet poorly understood, mechanisms allow meningococcal adhesion to cells of only human origin. Adhesion of encapsulated and virulent meningococci to human cells relies on the expression of bacterial type four pili (T4P) that trigger intense host cell signalling. Among the components of the meningococcal T4P, the concomitantly expressed PilC1 and PilC2 proteins regulate pili exposure at the bacterial surface, and until now, PilC1 was believed to be specifically responsible for T4P-mediated meningococcal adhesion to human cells. Contrary to previous reports, we show that, like PilC1, the meningococcal PilC2 component is capable of mediating adhesion to human ME180 epithelial cells, with cortical plaque formation and F-actin condensation. However, PilC1 and PilC2 promote different effects on infected cells. Cellular tracking analysis revealed that PilC1-expressing meningococci caused a severe reduction in the motility of infected cells, which was not the case when cells were infected with PilC2-expressing strains. The amount of both total and phosphorylated forms of EGFR was dramatically reduced in cells upon PilC1-mediated infection. In contrast, PilC2-mediated infection did not notably affect the EGFR pathway, and these specificities were shared among unrelated meningococcal strains. These results suggest that meningococci have evolved a highly discriminative tool for differential adhesion in specific microenvironments where different cell types are present. Moreover, the fine-tuning of cellular control through the combined action of two concomitantly expressed, but distinctly regulated, T4P-associated variants of the same molecule (i.e. PilC1 and PilC2) brings a new model to light for the analysis of the interplay between pathogenic bacteria and human host cells.

## Introduction

*Neisseria meningitidis* (Nme) is a strictly human pathogen that has two facets, a benign and a devastating one. Nme is carried by approximately 10% of healthy populations in Western countries, and up to 70% in military recruits [1]–[3]. Although carriage is most frequently observed, the fulminant forms of meningococcal infections break out unpredictably. The fulminant meningitis can kill previously healthy subjects within a few hours, making Nme one of the fastest killers of humans among known biological agents [4]. Meningococcal pathogenesis is a rare event that relies on the ability of the bacteria to break host defences such as cellular epithelial or endothelial barriers [5], [6]. The closely related pathogen *Neisseria gonorrhoeae* (Ngo) is the causative agent of a sexually transmitted disease and can also be responsible for disseminated forms of infection [7]. Ngo and Nme exhibit a high degree of genetic, structural and morphological similarity [8]–[10] but preferentially target different host organs, which suggests pathogenic *Neisseria* express specific determinants that allow attachment to precisely targeted host cell populations.

Meningococcal pathogenesis, as well as carriage, involves direct physical interactions of Nme with host cells. Nme is primarily an extracellular pathogen with a striking feature of microcolony formation on the surface of the infected cell [11]–[13]. Among neisserial virulence factors, type IV pili (T4P) appear to be the only bacterial attribute that allows efficient adhesion of capsulated bacteria to host cells [14]. T4P are robust thin filaments of up to 40 micrometers long that undergo dynamic cycles of assembly, exposure at the bacterial surface and retraction [15]. Pilus-mediated adhesion and filament retraction participate in a signalling system in which Nme is capable of modulating the host cell signalling machinery through T4P [16]. T4P-mediated adhesion induces cytoskeleton re-arrangements as well as modification of global intracellular signalling networks [17], [18]. Signalling is associated with the formation of “cortical plaques”, with dense actin polymerisation underneath bacterial clusters and accumulation of membrane-associated proteins such as ICAM-1, CD44 and EGFR (epidermal growth factor receptor) [19]. In human brain endothelial cells, T4P-mediated meningococcal adhesion leads to the formation of ectopic intercellular junctional domains at the site of bacteria host-cell interaction. This recruitment leads to the depletion of junctional proteins at the cell-cell interface and to the opening of the intercellular junctions [20]. Moreover, Nme evokes early intracellular calcium signalling during the course of infection, paralleled by MAPK pathway activation and interleukin release [21]. Cellular response to T4P-mediated infection varies among cell types. Membrane shedding in ME180 cells following gonococcal infection was shown to release CD46-enriched vesicles in the medium in a PilT-dependent manner, but such a phenomenon could not be observed with Hep-2 cells [22].

Besides adhesion, Nme can also enter host cells. For endothelial cells, internalisation relies on the activation of ErbB2, cortactin phosphorylation and activation of phosphoinositide-3-kinase signalling pathways [17], [18]. ErbB2 is a member of the EGFR family, which belongs to receptor tyrosine kinases. However, it is still unclear if similar signalling also exists in other cell types and little is known about the cellular motile response upon bacterial interaction during meningococcal infection.

Members of the EGFR family are membrane receptors involved in various cellular processes such as cell growth, proliferation and motility. Deregulation of EGFR was shown to be involved in the formation of multi-cellular aggregates *in vitro* [23]. Upon EGF binding to its specific membrane receptor at the cellular surface, EGFR undergoes dimerisation and auto-phosphorylation on multiple tyrosine residues, a key event in the activation of downstream signalling cascades. Following phosphorylation, the dimerised EGFR undergoes internalisation from the plasma membrane to subcellular locations, *via* both clathrin-dependent and independent endocytosis [24].

The PilC family of T4P-associated components is a major regulator of Nme adhesion and pilus retraction [15]. The PilC proteins enable pilus expression at the bacterial surface, transformation competence and adhesion to human cells [25]–[28]. Most pathogenic *Neisseria* express two PilC variants, which are independently expressed from separate loci and distinctly regulated [29], [30]. Both meningococcal PilC isoforms mediate bacterial piliation and transformation competence. However, only the PilC1 variant has been shown to be associated with meningococcal adhesion to human epithelial or endothelial cells [26], [31], [32]. Meningococci expressing solely the PilC2 protein were described as non-adherent despite their ability to form pilus structurally similar to those expressed in the presence of PilC1. Intriguingly, in the closely related gonococcus, both PilC proteins were shown to be functionally identical since they similarly promote piliation, transformation competence and adhesion to human cells [27]. Moreover, gonococcal PilC proteins have been described as adhesins, factors allowing attachment to the host cell [33]. The meningococcal PilC2 protein was thus considered as a defective variant of the PilC family that would promote piliation and transformation competence but not adhesion to host cells [26]. The molecular basis for the functional differences between meningococcal PilC1 and PilC2 variants could be associated with sequences specificities in the aminoterminal part of the protein [34].

In this work, we investigated the respective roles of the PilC family members in meningococcal attachment to ME180 epithelial cells. Our data show an unexpected role of the meningococcal PilC2 variant in efficiently mediating adherence of Nme to ME180 cells, a role thought to be restricted to PilC1. Intriguingly, the two meningococcal PilC variants trigger different cellular responses, affecting cellular motility and modulation of the EGFR signalling pathways.

## Results

### The meningococcal PilC2 variant enables bacterial adhesion to ME180 cells

**Figure 1:**
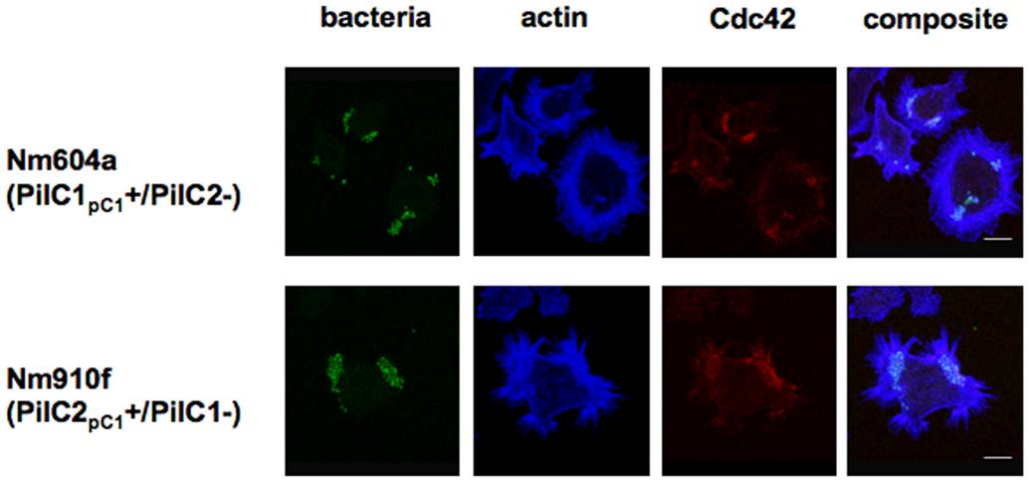
Cortical plaque formation following PilC1- or PilC2-mediated infection of ME180 cells. ME180 cells were infected for 15 minutes (MOI 100) with isogenic derivatives of the Nm2C4.3 wild type strain. Strains Nm604a and Nm910f respectively express solely either PilC1 or PilC2, under the control of the endogenous promoter of *pilC1*. Bar is 10 µm. Both strains trigger condensation of cellular F-actin at the site of bacterial attachment, as well as accumulation of Cdc42, suggesting intense and localised activation of cellular signalling pathways.

**Figure 2:**
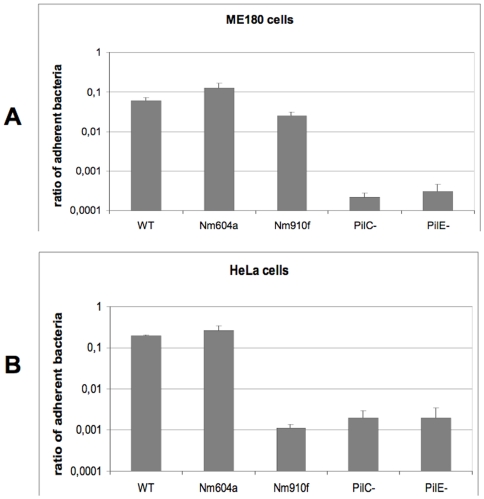
Adhesion of Nm2C4.3 derivatives to ME180 and HeLa cells. The ratio of adherent bacteria is expressed, on a logarithmic scale, as a ratio of bacteria adhering to the cellular monolayer to the total amount of infecting bacteria after 3 hours infection (MOI 100). Data represent the mean of 4 replicates of representative experiments +/− standard deviation of the mean. Bacterial strains are WT (Nm2C4.3, PilC1_wt_+/PilC2_wt_+), Nm604a (PilC1_pC1_+/PilC2-), Nm910f (PilC2_pC1_+/PilC1-), PilC- (PilC1-/PilC2-), PilE- (non piliated defective *pilE* strain). (A) ME180 cells; (B) HeLa cells. Two orders of magnitude separate adhesion rates of non-adherent from adherent strains, either on ME180 or HeLa cells. The meningococcal PilC2 variant mediates adhesion to Me180 but not to HeLa cells.

Until now, pilus-mediated meningococcal adhesion to eukaryotic epithelial or endothelial cells was specifically attributed to the expression of PilC1 [26]. Unexpectedly, we observed that meningococci lacking PilC1 were able to efficiently adhere to ME180 cells, which was in contrast with previous reports using the same cell line but other bacterial strains [32]. In order to verify if the PilC2 variant could indeed mediate the adhesion of meningococci to ME180 cells, we employed isogenic derivatives of the Nm2C4.3 strain solely expressing either PilC1 or PilC2 in similar amounts under the control of the endogenous promoter of *pilC1*, named Nm604a (PilC1_pC1_+/PilC2-) and Nm910f (PilC2_pC1_+/PilC1-) [34]. With this setting, the respective roles of each protein could be investigated independently from regulation specificities and this strategy also excluded the possibility of different amounts of PilC being a prime attribute for any observed cellular response. As for PilC1, we detected that PilC2-mediated adhesion to ME180 cells elicited cortical plaque formation [19], with intense F-actin condensation and clustering of signalling molecules such as the small GTPase Cdc42 (Figure 1). Comparison of adhesion levels with isogenic strains expressing PilC1 and/or PilC2, using poorly piliated or non piliated PilC/PilE null-mutated strains as negative controls, revealed the same order of magnitude as the wild-type (Figure 2), although PilC2-mediated adhesion to ME180 cells appeared slightly lower than PilC1-mediated adhesion. No systematic screening for human cell lines or primary cells was performed, but no significant adherence was observed for PilC1-/PilC2+ strains to other epithelial (HEC-1-B, HeLa) or endothelial (HUVEC or HBMEC) cell types, as previously reported [26], [32], suggesting stringent cellular specificity. Thus, we establish a first human cell culture model, namely the epithelial ME180 cells, for the quantitative analysis of meningococcal PilC2-mediated adhesion.

**Figure 3:**
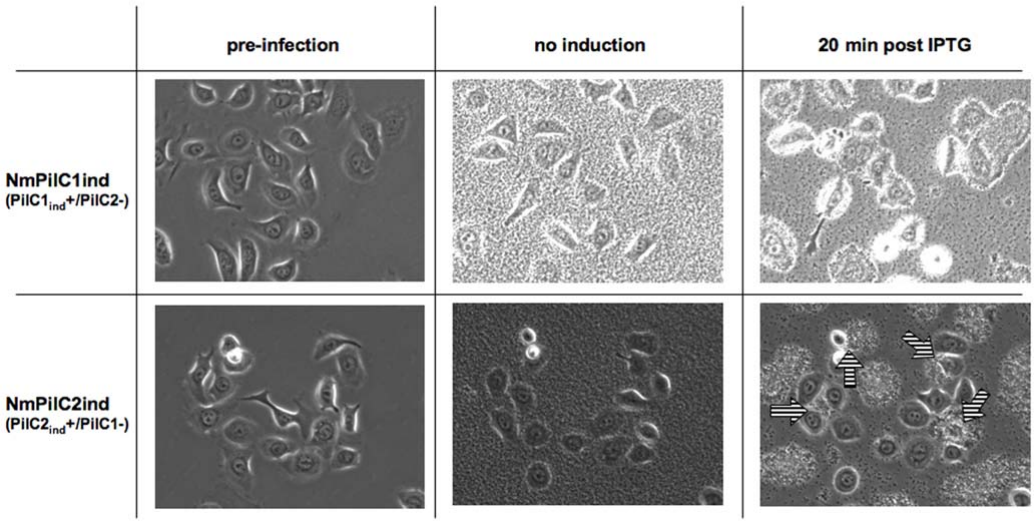
Adhesion monitoring of meningococci to ME180 cells upon IPTG-mediated PilC1 or PilC2 induction: Semi-confluent ME180 cells are monitored prior to (pre-infection) and during infection with Nm2C4.3 derivatives NmPilC1ind and NmPilC2ind that express virtually no PilC in the absence of induction. Infection (MOI 50) is initially carried out in the absence of IPTG for approximately 2 hours (no induction), and continued after addition of IPTG (20 min post IPTG). No meningococcal adhesion to ME180 cells is visible until expression of PilC1 or PilC2 is induced, despite heavy bacterial load due to replication over the experiment. Within minutes following addition of IPTG, all cells are covered with adhering bacteria in the case of PilC1-mediated infection, whereas only a fraction of ME180 cells are associated with PilC1-/PilC2+ meningococci (arrows).

Besides PilC, numerous bacterial factors are involved in the interaction of pathogenic *Neisseria* with human cells [6], [35]. In order to ascertain that PilC2 was specifically responsible for adhesion of PilC1-/PilC2+ meningococci to ME180 cells, we engineered isogenic strains solely expressing either PilC1 (NmPilC1ind) or PilC2 (NmPilC2ind) under the control of an IPTG inducible promoter, instead of endogenous promoters [15]. This allowed control of bacterial T4P expression and of adhesion through IPTG-controlled expression of PilC1 or PilC2 (Figure S1). Using live-cell microscopy, we monitored adhesion of these inducible strains to ME180 cells over time, starting in the absence of induction and followed by the addition of IPTG after 2 hours of bacteria-cell interaction (Figure 3, Video S1 and Video S2). In the absence of induction, the lack of expression of either PilC1 or PilC2 was associated with poor bacterial clumping (linked to defective piliation), and a lack of bacterial adhesion to cells despite the presence of a large bacterial load. In contrast, the expression of PilC1 or PilC2 within minutes of IPTG addition resulted in increased bacterial piliation and bacterial adhesion to the cells (Figure 3, 20 min post IPTG). As previously described [15], bacterial clustering into microcolonies and twitching upon PilC induction is consistent with an increase in bacterial expression of functional T4P. The observation of efficient bacterial adhesion to the host cell through IPTG-controlled expression of either PilC1 or PilC2 is coherent with data obtained with strains Nm604a and Nm910f that constitutively express PilC1 or PilC2, and demonstrates that, like PilC1, the meningococcal PilC2 can specifically mediate bacterial adhesion to ME180 cells.

### PilC1 and PilC2 trigger different cellular responses in ME180 cells

Although both constitutively expressed PilC1 and PilC2 variants promoted adhesion to ME180 cells and triggered apparently similar cortical plaque formation (Figure 1), live-imaging experiments using IPTG-inducible expression of PilC1 or PilC2 (Figure 3, Video S1 and Video S2) revealed that both strains elicited strikingly different dynamic cell responses upon infection. First, only a fraction (ca 30% to 50%) of ME180 cells were permissive to PilC2-mediated adhesion whereas numbers were close to 100% for PilC1. Second, host cell motility differed after infection mediated by each variant since bacterial adhesion mediated through the induction of PilC1 led to reduced cell migration, while the induction of PilC2 triggered no apparent change in the motility of these cells upon adhesion. These observations suggested mechanistic differences in the host cellular response elicited upon PilC1- versus PilC2-mediated adhesion.

**Figure 4:**
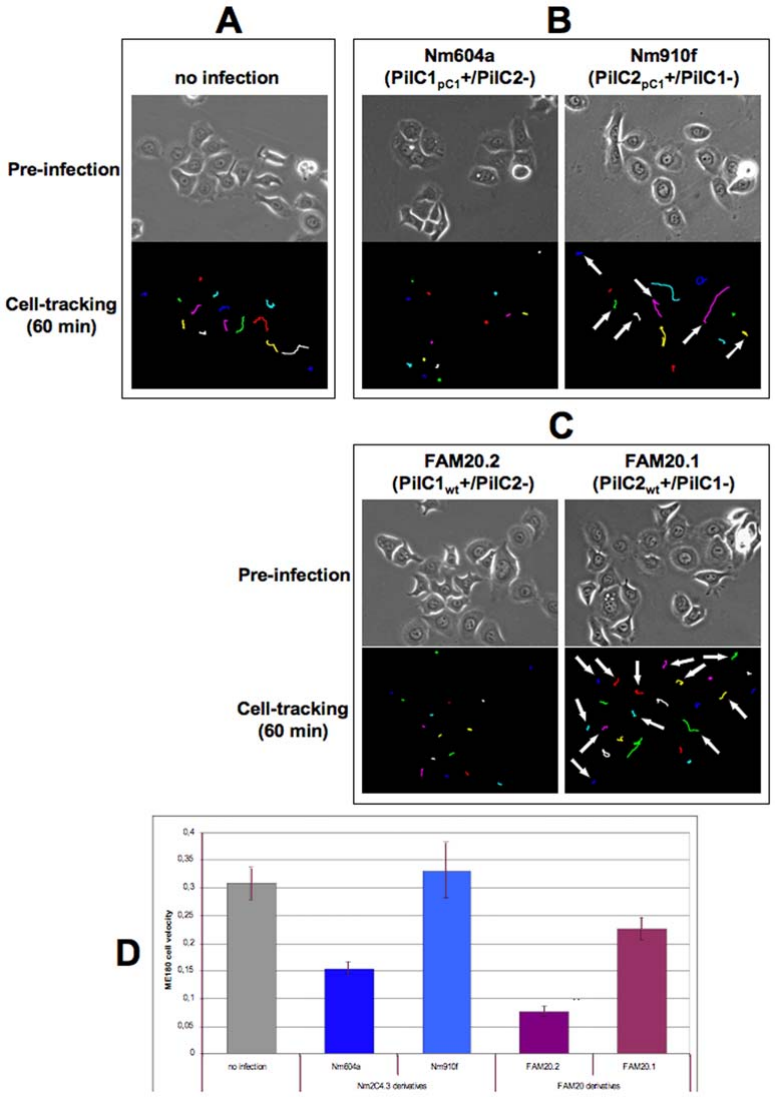
Cell-tracking analysis of ME180 cells upon PilC1- or PilC2-mediated infection. ME180 cells were infected (MOI 50) with strains Nm604a or Nm910f that express constitutively either PilC1 or PilC2, and tracked throughout the experiment. Cell monitoring was started before infection and continued throughout the experiment. In each panel, the upper image shows the cells prior to infection, and the lower image the cellular path after 60 min tracking. A: Non-infected cells. B: Infection with the Nm2C4.3 derivative strains Nm604a (PilC1_pC1_+/PilC2-) and Nm910f (PilC2_pC1_+/PilC1-). C: Infection with the FAM20 derivative strains FAM20.2 (PilC1_wt_+/PilC2-) and FAM20.1 (PilC2_wt_+/PilC1-). Virtually all cells were infected by PilC1-expressing strains Nm604a or FAM20.2. For the cells infected with strains Nm910f or FAM20.1, arrows indicate tracks corresponding to cells susceptible to PilC2-mediated adhesion, whereas cells that remained not infected in the course of the experiment are unmarked. D: Comparison of cellular velocity over 60 min following infection with either Nm2C4.3 derivatives (Nm604a and Nm910f) or FAM20 derivatives (FAM20.2 and FAM20.1), using non-infected cells as control (in µm/min). Data represent the average velocity for all cells in the field, +/− standard error of the mean. For both series of mutants derived from unrelated strains Nm2C4.3 and FAM20, PilC1-mediated infection is specifically associated with a decrease in cellular motility, whereas cells infected through PilC2 remain motile.

In order to further investigate the role of each PilC variant in host cell motility independently from possible artefacts caused by IPTG induction, we used time-lapse microscopy to monitor PilC1- or PilC2-mediated adhesion of the meningococcal isogenic Nm604a and Nm910f strains to ME180 cells. These strains, already used in Figure 1 and Figure 2, express similar amounts of either PilC1 or PilC2 proteins through the control of the endogenous promoter of *pilC1* [34]. Infection of ME180 cells with these strains (Figure 4) elicited similar dynamic and segregation phenotypes to those observed under conditions of PilC1 or PilC2 induction with IPTG. Cellular tracking analysis revealed that meningococci expressing *pilC1* under the control of its endogenous promoter caused a severe reduction of cellular motility upon infection, whereas infection with Nme expressing *pilC2* under the control of the same promoter failed to trigger a significant alteration in ME180 motility throughout the experiment (Figure 4, B and D).

Besides altering motility, PilC1-mediated infection was eventually associated with loosening of cellular attachment to the substratum that could be detected within the first hour of infection, and with formation of infected cell aggregates. In contrast, cellular attachment to the substratum was not altered during PilC2-mediated infection. This phenomenon was seen using a meningococcal strain with an IPTG-inducible promoter for *pilC1* or *pilC2* (Video S1 and Video S2), as well as with non-inducible promoters. Since cell detachment from the substratum is a trait of apoptosis, we investigated if PilC1-mediated infection would lead to chromatin condensation, a signature of apoptosis [36]. No significant chromatin condensation and/or nucleus fragmentation could be observed up to 6 hours after infection (data not shown). Together with the rapid change in cellular motility and cell-to-substratum adhesion, the data ruled out the possibility that apoptosis had a major role in PilC1-mediated early detachment of ME180 cells from the substratum.

To extend our observations, we performed the same experiments using FAM20, another previously described pathogenic meningococcal strain that is unrelated to Nm2C4.3 and its derivatives. The FAM20 strain belongs to serogroup C and attachment of this strain to ME180 cells was reported to be facilitated by PilC1 but not PilC2 [32]. Analysis of the previously described FAM20.2 (PilC1_wt_+/PilC2-) or FAM20.1 (PilC1-/PilC2_wt_+) derivatives of the FAM20 strain [32] in time-lapse infection experiments with ME180 cells showed patterns of adhesion similar to those observed with the Nm604a and Nm910f derivatives of Nm2C4.3; FAM20.1 efficiently adhered to a fraction of ME180 cells, and FAM20.2 triggered a severe reduction in the motility of the infected cells (Figure 4, C and D). Thus, the ability of both PilC1 and PilC2 meningococcal variants to mediate adhesion and a specific host cell response is observed for unrelated meningococcal strains.

As already mentioned, IPTG-induced PilC1-expressing meningococci adhered to virtually all cells present, while only a subset of cells in a ME180 monolayer was permissive to PilC2-mediated adhesion (Figure 3). To investigate if different ME180 clonal cell populations could be responsible for the heterogeneity of PilC2-mediated adhesion, we analysed an array of clonal cell subsets, each clone being generated from a single ME180 cell. Using Nm604a (PilC1_pC1_+/PilC2-) and Nm910f (PilC2_pC1_+/PilC1-) strains that constitutively express either PilC1 or PilC2, the proportion of cells permissive to PilC1- or PilC2-mediated infection among clones of ME180 cells was similar to that of the parental population of cells (Figure S2). Thus, differences in cellular susceptibility to PilC2-mediated infection are not restricted to clonal populations among ME180 cells, but more likely due to a particular, yet undefined, metabolic state of the cell in which a cellular receptor(s) for PilC2 is expressed.

These results show that both meningococcal PilC1 and PilC2 variants mediate specific host cell responses upon adhesion. Together with the fractional response of ME180 cells to PilC2-mediated infection, our data suggest that both PilC variants might operate via distinct, yet unknown, cellular receptors for piliated Nme.

### PilC1-mediated infection is specifically associated with EGFR degradation

**Figure 5:**
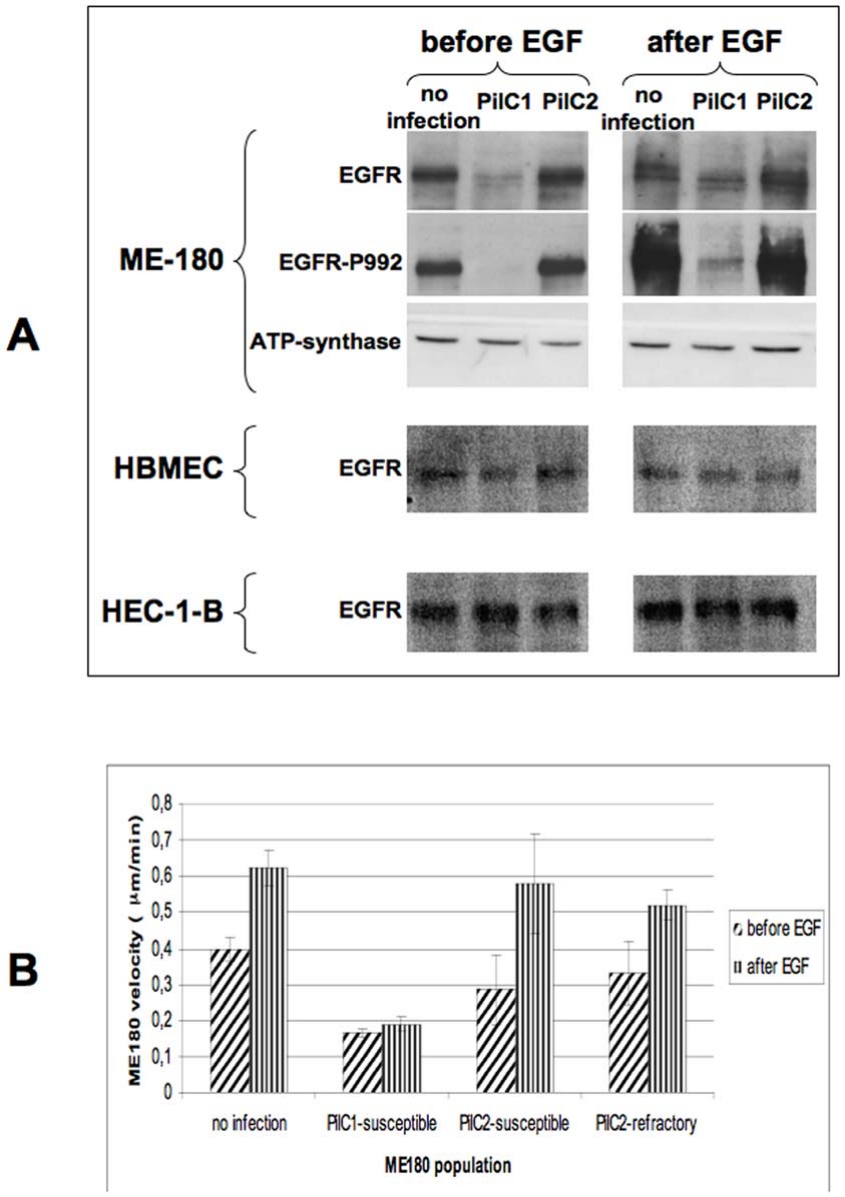
EGFR status upon PilC-mediated infection. Panel A: ME180, HBMEC or HEC-1-B cells were infected (MOI 100) with strains Nm604a (PilC1_pC1_+/PilC2-) or Nm910f (PilC2_pC1_+/PilC1-) for 2 hours before cells were stimulated with EGF (25 ng/ml, 5 min). Non-infected cells (no infection) were used as control. Cellular extracts were probed in western blot analysis for the presence of all forms of EGFR (EGFR), for a phosphorylated form of the receptor (EGFR-P992), or for ATP-synthase as a marker for the protein load of cellular extracts. Cells were collected just prior to addition of EGF (left panel), or 5 min after EGF stimulation (right panel). Exposure time of western blot was extended until signal was detectable in all lanes. Panel B: cells infected for 2 hours with either Nm604a (PilC1_pC1_+/PilC2-) or Nm910f (PilC2_pC1_+/PilC1-) were monitored using time-lapse microscopy for 30 min immediately before and after EGF stimulation (25 ng/ml). In the case of PilC2-mediated infection, tracking data were analysed separately for ME180 cells susceptible or refractory to infection. Data represent average velocity for all cells in the field in µm/min, +/− standard error of the mean.

Modulation of EGFR signalling through the RAF-MEK-ERK pathway has been shown to regulate cell adhesion, formation of cell aggregates and cellular motility [23]. Therefore, we investigated whether EGFR signalling was affected upon PilC1- or PilC2-mediated infection. ME180 cells were infected for 2 hours with either Nm604a (PilC1_pC1_+/PilC2-) or Nm910f (PilC2_pC1_+/PilC1-), and the level of EGFR was investigated. We observed a dramatic decrease in the amount of EGFR for PilC1-infected cells, in comparison to non-infected cells (Figure 5A, left panel-). This was observed as soon as 15 min post infection (data not shown). In contrast, PilC2-mediated infection did not notably affect EGFR levels in the host cells despite binding efficiently.

To investigate if modulation of EGFR levels would be the reflect of functional properties, we analysed the phosphorylation status of EGFR in response to EGF stimulation of infected ME180 cells, using non-infected cells as a control. The same experimental protocol was used but cells were additionally stimulated with EGF for 5 min at the end of the 2-hour infection, prior to collection of cellular material and western blot analysis (Figure 5A, right panel-). Similarly to non-infected cells, PilC2-mediated infection left ME180 cells permissive for EGFR phosphorylation upon EGF stimulation. In contrast, EGF stimulation of cells infected with a PilC1+ strain resulted in a weak phosphorylation of EGFR. Because the total protein level of EGFR prior to EGF stimulation was low in cells infected through PilC1 (Figure 5A, left panel-), the strong reduction in EGFR phosphorylation was probably due to the depletion of the total EGFR pool upon PilC1-mediated infection. RT-PCR analysis detected no notable change in EGFR mRNA expression during the course of the experiment, suggesting that EGFR degradation and recycling pathways are differently involved upon PilC1- or PilC2-mediated adhesion. Similar results were observed for the phosphorylated forms of ErbB2 (data not shown). Figure 5-A also shows that PilC1-mediated adhesion of meningococci to other cell types such as HBMEC or HEC-1-B did not result in the depletion of the EGFR cellular pool and supports previous reports on EGFR clustering under meningococcal microcolonies [19].

We further analysed the response of ME180 cells to EGF during infection with meningococci expressing constitutively PilC1 (Nm604a) or PilC2 (Nm910f), by monitoring cell motility for 30 minutes just prior to, or just following, addition of EGF to the cells. Figure 5-B shows that non-infected ME180 cells respond to EGF stimulation by increasing motility. Consistent with western blot analysis of EGFR phosphorylation, we found that PilC1-infected cells were poorly responsive to EGF activation. In the case of PilC2-mediated infection, EGFR phosphorylation response to EGF stimulation was slightly lower in comparison to non-infected control cells. We therefore investigated if this difference could be due to fractional susceptibility of ME180 cells by separately analysing ME180 cells susceptible or refractory to PilC2-mediated adhesion. We observed that PilC2-mediated infection did not alter the motility response to EGF, neither for cells associated with bacteria nor for cells devoid of attached meningococci. Thus, our results suggest that PilC1 is specifically responsible for the depletion of the EGFR pool in ME180 cells upon infection.

Taken together, our results show that both meningococcal PilC1 and PilC2 variants mediate specific adhesion to ME180 cells, but trigger different cellular responses for cellular motility and signalling affecting EGFR pathways. The difference in host cell response is associated with the expression of closely related pilus-associated components that are independently regulated in wild type strains, thus presenting a new and intriguing model for studying the modulation of the eukaryotic response to infection by T4P-expressing pathogens.

## Discussion

In this study, we investigated how meningococcal infection differentially modulates host cell motility and EGFR signalling pathways through two independently regulated variants of T4P components. Infection of human cells by pathogenic *Neisseria* is a complex process that involves bacterial attachment to the eukaryotic cell and intracellular signalling. In the case of Nme, T4P play a central role since they are the only attributes allowing adhesion of capsulated bacteria to cells and are present in most, if not all, clinical bacterial isolates [14]. These events are associated with the formation of “cortical plaques” at the site of bacterial attachment, where numerous components of actin microfilaments and signalling molecules are recruited [19]. Our results show that both meningococcal variants of the T4P-associated PilC component, PilC1 and PilC2, are capable of mediating adhesion independently. Moreover, these two proteins, which are concomitantly expressed but distinctly regulated in wild type strains of Nme, elicit different structural and signalling responses in the host cell.

Our observation that the PilC2 variant of the meningococcal FAM20 strain promotes adhesion to ME180 cells differs from previous work [32]. However, technical points may account for the differences in phenotype observed here. First, Rahman et al. measured bacterial adhesion by optical evaluation of bacterial counts on 50 cells. In our experiments, optical numeration of cell-associated diplococci appeared poorly reproducible since attaching meningococci form three-dimensional aggregates of various size and shape. Instead, we calculated adhesion ratios comparing the number of CFU recovered from cell-associated bacteria to the total amount of bacteria in the well, at the corresponding time-point. Second, figures in previous report were obtained using non-confluent ME180 cells, whereas adhesion ratios reported here were obtained with cells at higher densities (sub-confluent). Moreover, the fractional susceptibility of ME180 cells to PilC2-mediated adhesion may have artificially lowered the average bacterial count per cell. Third, alteration of phenotype might be due to phase variation in the expression of PilC1 or PilC2 in the FAM20 derivatives, since FAM20.1 and FAM20.2 were obtained by simple cassette-mutagenesis and carry endogenous *pilC* promoters. For this reason, we used Nm2C4.3 derivatives with engineered *pilC* promoters that prevent phase variation and allow identical regulation for both *pilC* variants. Forth, both unrelated Nm2C4.3 and FAM20 strains express strain-specific PilC1 or PilC2 variants with different primary structures. Last, dynamic aspects of meningococcal adhesion might also be involved. The regulation of both *pilC1* and *pilC2* genes was shown to be drastically different in the Nm2C4.3 strain [30] but was not investigated in the FAM20 strain. Taken together, the FAM20.1 and FAM20.2 strains express PilC1 or PilC2 variants with specific primary structures and with uncharacterised promoters. For these reasons, our analysis was focused on the isogenic Nm2C4.3 derivatives, Nm604a and Nm910f, which express either *pilC1* or *pilC2* with identical regulation. The observation that, in live-imaging experiments, unrelated meningococcal strains with endogenous *pilC* promoters (i.e. FAM20.1 and FAM20.2) lead to results similar to those obtained with promoter-engineered strains (i.e. Nm2C4.3 derivatives) strengthens our conclusions on the respective roles of PilC1 and PilC2 in the motility control of infected human cells.

Among different epithelial (HeLa, HEC-1-B) or endothelial (HUVEC, HBMEC) cell types tested, the endometrium-derived ME180 was the only cell line that was permissive for PilC2-mediated adhesion. The endometrium is unlikely to play a central role in the pathogenesis of Nme, but restriction of Nme PilC2-mediated adhesion to ME-180 cells could indicate a highly specific modulation of meningococcal cellular binding to yet unrecognised cell types of the nasopharynx. Although we did not investigate primary cell types isolated from the nasopharynx for this phenotype, our results show that human cells (i.e. ME180) can express a cellular receptor(s) for PilC2. This suggests that meningococci have evolved a highly discriminative tool for differential adhesion in specific microenvironments where different cell types are present.

Both meningococcal PilC variants promote cortical actin rearrangements upon adhesion to ME180 cells but we show that only PilC1 induces reductions in EGFR levels and motility, thus suggesting complementary functions for both variants. Based on these results, the meningococcal PilC2 variant should no longer be considered as a defective variant for adhesion, but as a functional variant with specificities for restricted cell types. Therefore, the independent regulation of both PilC variants in wild type bacteria could enable meningococci to sequentially modulate host cell response in a controlled manner, with partially overlapping (i.e. cellular binding) and partially antagonising (i.e. depletion of EGFR pool and motility) effects, depending on the cellular diversity of each ecological niche. The hypothesis of selective PilC-mediated modulation of cellular response is emphasized by the observation that, although being closely related, both gonococcus and meningococcus show highly specific tropism for host tissue. In the genetically related gonococcus, both variants of PilC have been described as mutually replaceable [9], [31] but the differential regulation of both genes was not investigated. Further work is thus needed to decipher (i) if, beside adhesion to host cells, gonococcal PilC1 and PilC2 variants promote qualitatively different host cell responses, and (ii) how the regulation of both *pilC* genes is in control of the cellular response. The future development of experimental models for the ecological niches of Nme and Ngo, involving different cell types as well as the extracellular matrix, will help to shed new light on this central, but poorly investigated, aspect of neisserial interaction with the human host.

The different responses elicited by meningococcal infection among various cell types can be regarded as a hallmark of neisserial infection. Our data showing EGFR degradation in ME180 cells upon meningococcal infection contrast with previous data on gonococcal infection of HEC-1-B and A431 cells showing EGFR accumulation [19]. Specificities in cell-type response to neisserial infection was also described for other cellular pathways such as membrane shedding upon gonococcal infection, which is seen with ME180 cells but could not be observed on Hep2 cells [22]. Only a fraction of ME180 cells are susceptible to PilC2-mediated infection whereas virtually all cells are infected through PilC1. We hypothesised that a particular physiological state of the cell would be responsible for this phenomenon. Although our search for a link with the cell cycle was unconclusive, recent work from other groups has shown that gonococcal infection of epithelial cells, including ME180 cells, is increased for cells in interphase (G1, S or G2) rather than in M or G0 of the cell cycle [37].

Taken together, we show that, unlike PilC1 that enables meningococcal attachment to many epithelial or endothelial cells, the meningococcal PilC2 protein selectively mediates adhesion to restricted cell types. Moreover, the different cellular response mediated by PilC1 and PilC2, combined with the independent regulation of both variants of the same protein, suggests a new model for the fine-tuning of host cell behaviour by the meningococcus during infection.

## Materials and Methods

### Bacterial strains and media

Nm2C4.3 is a derivative of Nme strain 8013, a serogroup C, class 1 strain [38]. This strain is piliated and adherent to human cells, Opa-, Opc-, PilC1+ and PilC2+. *Neisseria* were grown at 37°C in a 5% CO2 atmosphere on GC medium (Difco-BD, NJ, USA) containing Kellogg's supplement [39]. For selection of meningococcal strains, kanamycin was used at a concentration of 100 µg/ml, erythromycin at 2 µg/ml, chloramphenicol at 10 µg/ml and tetracycline at 1 µg/ml. The PilE-defective mutant (PilE-) of Nm2C4.3 has been previously described [11], as well as the PilC-null (PilC-) derivative [34]. Nm604a (PilC1_pC1_+/PilC2-) and Nm910f (PilC2_pC1_+/PilC1-), the isogenic derivatives of the Nm2C4.3 strain expressing solely either *pilC1* or *pilC2* under the control of the endogenous *pilC1* promoter, showing an inactivated wild type *pilC2* locus (*pilC2::ermAM*) and harbouring an *aphA3* kanamycin resistance cassette downstream of the expressed *pilC* gene, were previously described and allowed to investigate the respective role of each protein independently from regulation specificities [34]. Piliation and sequence of pilin gene, expression of capsule and absence of both Opa and Opc were verified. Unrelated to strain 8013, the FAM20 meningococcal strain is piliated, expresses both PilC1 and PilC2 and belongs to capsular serogroup C. The meningococcal FAM20 derivatives FAM20.1 (PilC1-/PilC2_wt_+) and FAM20.2 (PilC1_wt_+/PilC2-) were previously described [32] and kindly provided by A.B. Jonsson. FAM20.1 and FAM20.2 are cassette mutagenesis defective mutants that express either *pilC1* or *pilC2* under the control of their respective endogenous promoters, in contrast to Nm604a and Nm910f derivatives of the Nm2C4.3 strain that express *pilC1* or *pilC2* under the control of an identical promoter. They were restreaked on chloramphenicol-containing agar GC plates and no additional engineering was performed on these strains.

### Construction of strains harbouring inducible *pilC* genes

Meningococcal derivatives of Nm2C4.3 expressing IPTG-inducible *pilC1* and *pilC2* genes were engineered as previously described for a *pilC1* inducible strain, allowing tight control of PilC expression [15], with the difference that both recombinant PilC variants carried a 6-HIS tag at the amino-terminal end of the mature protein. Briefly, the previously described Nm2C4.3 derivatives Nm604a and Nm910f, expressing either *pilC1* or *pilC2*, were used for the construction of *pilC*-inducible strains. The endogenous *pilC1* promoter region was replaced by an IPTG-inducible promoter (gift of H. S. Seifert), carrying a tetracycline-resistance cassette together with the 5′-moiety of either *pilC1* or *pilC2*. Oligonucleotides used for inserting the region coding for a 6-His tag at the amino-terminal end of the mature PilC protein were: C1N-HIS-APA (5′-GGG CCC AGG CGCA AAC CCA TCA CCA CCA TCA TCA CAG TAA ATA CGC TAT TAT CAT GAA CGA A-3′), C2N-HIS-APA (5′-GGG CCC AGG CGC AAA CCC ATC ACC ACC ATC ATC ACA ACA CCT ATC CAT ACG TTA TTG TAA TG-3′), and CR328BsiWI (5′- GAA ACC TTG CCG TAC GGC GGC AGG TAG GT-3′). Constructs were made in *E. coli* and subsequently introduced in Nme using natural transformation competence and selection of transformants with erythromycin, kanamycin and tetracycline, as the concentrations listed above. Under conditions of IPTG induction, both resulting strains NmPilC1ind (PilC1_ind_+/PilC2-) and NmPilC2ind (PilC2_ind_+/PilC1-) exhibited phenotypes similar to those of the corresponding mother-strains expressing solely one PilC variant, and the nucleotide sequence of each *pilC* gene was verified. Similarly to previously described strains [15], virtually no expression of PilC was detected in the absence of induction. The T4P-related phenotypes (piliation and adhesion level to epithelial cells) of the resulting NmPilC1ind and PilC2ind strains are shown in Figure S1.

### Cell culture and adhesion assays

The cell lines used in the experiments were ME180 human cervix carcinoma (ATCC HTB-33), HeLa human cervix carcinoma (ATCC CCL-2), human uterus endometrium adenocarcinoma HEC-1-B cells (ATCC HTB-113) and human bone-marrow endothelial HBMEC cells. The ME180 cells were maintained in McCoy's 5A medium supplemented with L-glutamine and 10% FCS. HeLa cells were cultured in RPMI-1640 medium supplemented with 2 mM L-glutamine and 10% FCS. HEC-1-B cells were cultured in minimum essential medium supplemented with 2 mM L-glutamine, 0.1 mM non essential amino acids, 1 mM sodium pyruvate and 10% FCS. HBMEC cells [40], [41] were kindly provided by C. R. Hauck (Zentrum für Infektionsforschung, Universität Würzburg, Würzburg, Germany), and cultured in DMEM Glutamax supplemented with 10% FCS, 7.5 µg/ml endothelial-cell growth supplement (Sigma), 7 IU heparin and 10 mM Hepes (pH 7.4) on gelatin-coated plates. Cells were grown at 37°C in a humidified incubator under 5% CO_2_.

For adhesion counts, cells were grown in 24-well plastic cell culture dishes to sub-confluency. Monolayers were washed with medium and bacteria were added to the cells at a multiplicity of infection (MOI) of 100, as for other end-point adhesion experiments. Infection was performed in medium without FCS. Infected and non-infected monolayers were centrifuged for 3 min at 120 g to synchronise infection, and incubated at 37°C in 5% CO2. At the end of incubation time (up to 3 hours depending on experiment), infection was stopped and non-adherent bacteria were removed by washing the cells three times with medium. Cell-associated bacteria were quantified after cell lysis with 1% saponin in medium. Colony forming units (CFU) were determined by plating serial dilutions.

For immunofluorescence experiments, cells were cultured on collagen-coated glass coverslips and infected as described for adhesion counts. At the end of infection time, infected cells were rinsed three times with PBS to remove non-adherent bacteria and immediately fixed with 3.7% paraformaldehyde (PFA), before immunostaining.

### Live cell-imaging

Live imaging adhesion assays were performed with ME180 cells grown in 35 mm cell-culture plastic dishes (BD Falcon, Bedford, MA, USA). One day prior to the experiment, cells were seeded at a density of approximately 1×10^4^ cells/cm^2^ (30–50% confluency). Three hours before infection, culture medium was replaced by warm RPMI medium. Infection was performed with freshly grown bacteria resuspended in RPMI medium with sufficient time given (10–30 min) so that piliated bacteria displayed twitching activity. Microscopic detection of twitching activity was deemed an indicator of the establishment of bacteria-cell interaction. Cell monolayers were infected at an MOI of 50 since incubation was carried out for up to 5 hours without washing nor disturbing infection of the monolayer. Time-lapse live imaging was performed with a ZEISS Axiovert 200 microscope, using a 40x objective. Imaging was performed with a time lapse of 30 seconds throughout the experiment, which allowed tracking of individual cells before and throughout infection. Addition of bacteria to the medium interrupted cell monitoring for less than one minute. In order to ensure minimal interference with cellular adhesion and migration processes, ME180 cells were devoid of any plasmid constructs and experiments were all made using the same brand of dishes. For adhesion experiments with strains carrying inducible *pilC* constructs, 1 mM IPTG was added to the bacteria after approximately 2 hours of established bacteria-cell interaction, and maintained until the end of the experiment.

### Image analysis

Image analysis was performed using the ImageJ software and the plug-in “Manual Tracking” from Fabrice Cordelières, Institut Curie, Orsay (France). For velocity analysis, results are expressed in µm/min.

### Proteins and immunoblotting

For Western blot analysis, antibodies recognising the following proteins were used (1∶1000 dilution): phospho-EGF receptor (Tyr-992) (Cell Signaling 2235), EGF receptor (Cell Signaling 2232) and ATP synthase (BD Biosciences 612516). Cell lysates were resolved by 10% sodium-dodecyl-sulfate (SDS)-poly-acrylamide gel electrophoresis and transferred to poly-vinylidene difluoride membranes. After blocking with Tris-buffered saline containing 0.1% Tween 20 and 5% non-fat dry milk, membranes were probed with specific antibodies. Proteins were visualised with peroxidase-coupled secondary antibody (1∶1000 dilution) using the ECL system (Amersham).

### Immunofluorescence of infected cells

After 5 min fixation with 3.7% PFA, cells fixed to glass coverslips were treated as previously described [34]. A Phalloidin-A635 probe (Molecular Probes) and primary antibodies recognising the following antigens were used (1∶1000 dilution): Cdc42 (Santa Cruz SC8401) or meningococci (rabbit anti-“Rou” serum recognising the whole bacteria).

## Supporting Information

Figure S1 Phenotypes associated with PilC1 or PilC2 induction in *N. meningitidis*. T4P-associated phenotypes (adhesion to human cells and piliation) were investigated for meningococcal strains upon induction of either PilC1 (strain NmPilC1ind) or PilC2 (strain NmPilC2ind) with IPTG. Control strains are wild-type Nm2C4.3 (WT) and, respectively, non/poorly piliated defective PilE/PilC mutants. A: Adhesion to either ME180 or HeLa cells was measured after 1-hour incubation (MOI 100) in the presence of IPTG concentrations ranging up to 1 mM (representative experiment). Meningococcal adhesion to ME180 cells was dependent on either PilC1 or PilC2 expression. However, as expected, adhesion to HeLa cells was exclusively restricted to PilC1-expressing strains. B: Liquid-grown bacteria were tested for the presence of pili after 1H of induction with IPTG, using a polyclonal anti-pilin antibody. For both NmPilC1ind and NmPilC2ind strains, piliation correlates with IPTG-controlled induction of PilC1 or PilC2.
(0.45 MB TIF)

Figure S2 Clonal permissivity of ME180 cells to PilC1- or PilC2-mediated infection. Adhesion assays with either PilC1- or PilC2-expressing meningococci (strains Nm604a and 910f, respectively) were performed on 17 (a–q) single-cell derived ME180 populations (MOI 100). For each cellular clone, the percentage of cells permissive to infection is indicated for each meningococcal strain. Controls are early (p-4) and late (p-26) passages of ME180 cells, as well as two populations derived from 100 pooled ME180 single cells (100-A and 100-B). No difference was observed for single-cell derived populations and controls.
(0.10 MB TIF)

Video S1 Live-imaging of ME180 cells infected with strain NmPilC1ind. ME180 cells were infected with meningococcal strains NmPilC1ind (PilC1ind/PilC2-) that expresses *pilC1* under the control of an IPTG-inducible promoter. Cells were monitored continuously before infection (in the absence of IPTG), after addition of infecting bacteria (MOI 50, no IPTG), and after the induction of PilC1 expression by addition of IPTG to the medium. Adhesion to ME180 cells relies on the expression of PilC1, upon IPTG-mediated induction. However, cellular motility is reduced in the case of PilC1-mediated infection, whereas it remains unaffected in the case of PilC2-mediated adhesion (Video S2).
(1.66 MB AVI)

Video S2 Live-imaging of ME180 cells infected with strain NmPilC2ind. Me180 cells were infected with meningococcal strain NmPilC2ind (PilC2ind/PilC1-) that expresses *pilC2* under the control of an IPTG-inducible promoter. Cells were monitored continuously before infection (in the absence of IPTG), after addition of infecting bacteria (MOI 50, no IPTG), and after the induction of PilC expression with addition of IPTG to the medium. Adhesion to ME180 cells relies on the expression of PilC2, upon IPTG-mediated induction. However, although cellular motility is reduced in the case of PilC1-mediated infection (Video S1), it remains unaffected in the case of PilC2-mediated adhesion.
(1.56 MB AVI)
