# Supplementary figures and images for: Neisseria meningitidis Differentially Controls Host Cell Motility through PilC1 and PilC2 Components of Type IV Pili
Source: PLoS One. 2009 Aug 31;4(8):e6834. doi: 10.1371/journal.pone.0006834 (PMC2729722; doi:10.1371/journal.pone.0006834)

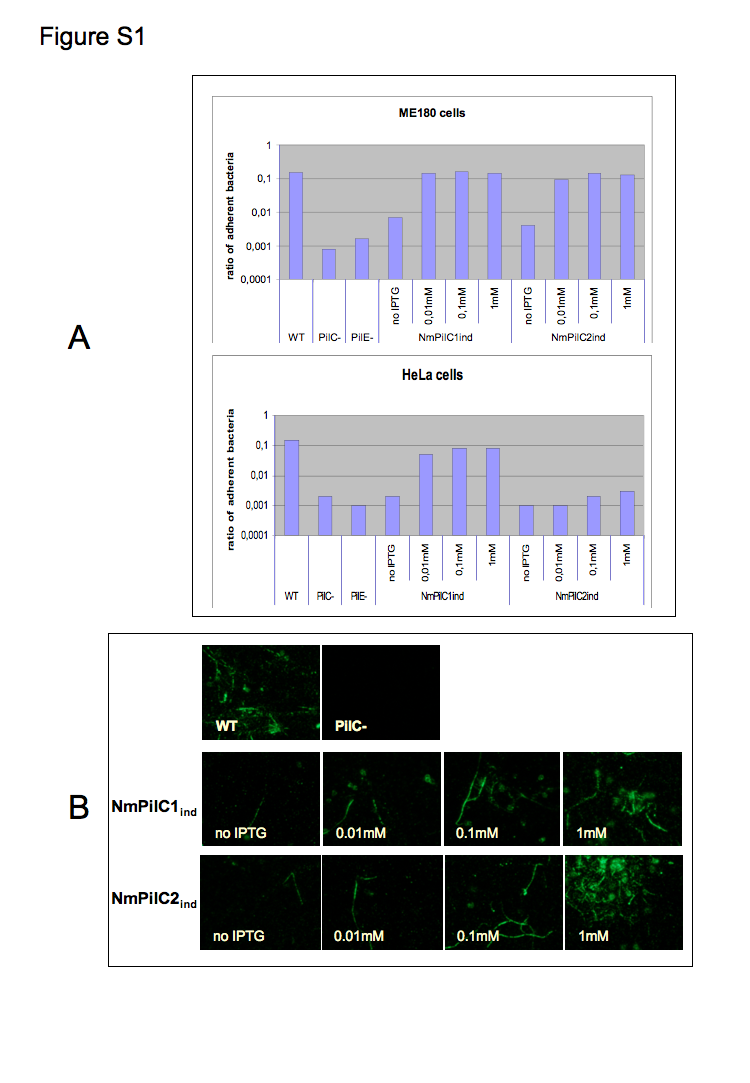

Supplement: Figure S1 — Phenotypes associated with PilC1 or PilC2 induction in N. meningitidis. T4P-associated phenotypes (adhesion to human cells and piliation) were investigated for meningococcal strains upon induction of either PilC1 (strain NmPilC1ind) or PilC2 (strain NmPilC2ind) with IPTG. Control strains are wild-type Nm2C4.3 (WT) and, respectively, non/poorly piliated defective PilE/PilC mutants. A: Adhesion to either ME180 or HeLa cells was measured after 1-hour incubation (MOI 100) in the presence of IPTG concentrations ranging up to 1 mM (representative experiment). Meningococcal adhesion to ME180 cells was dependent on either PilC1 or PilC2 expression. However, as expected, adhesion to HeLa cells was exclusively restricted to PilC1-expressing strains. B: Liquid-grown bacteria were tested for the presence of pili after 1H of induction with IPTG, using a polyclonal anti-pilin antibody. For both NmPilC1ind and NmPilC2ind strains, piliation correlates with IPTG-controlled induction of PilC1 or PilC2. (0.45 MB TIF) [file pone.0006834.s001.tif]

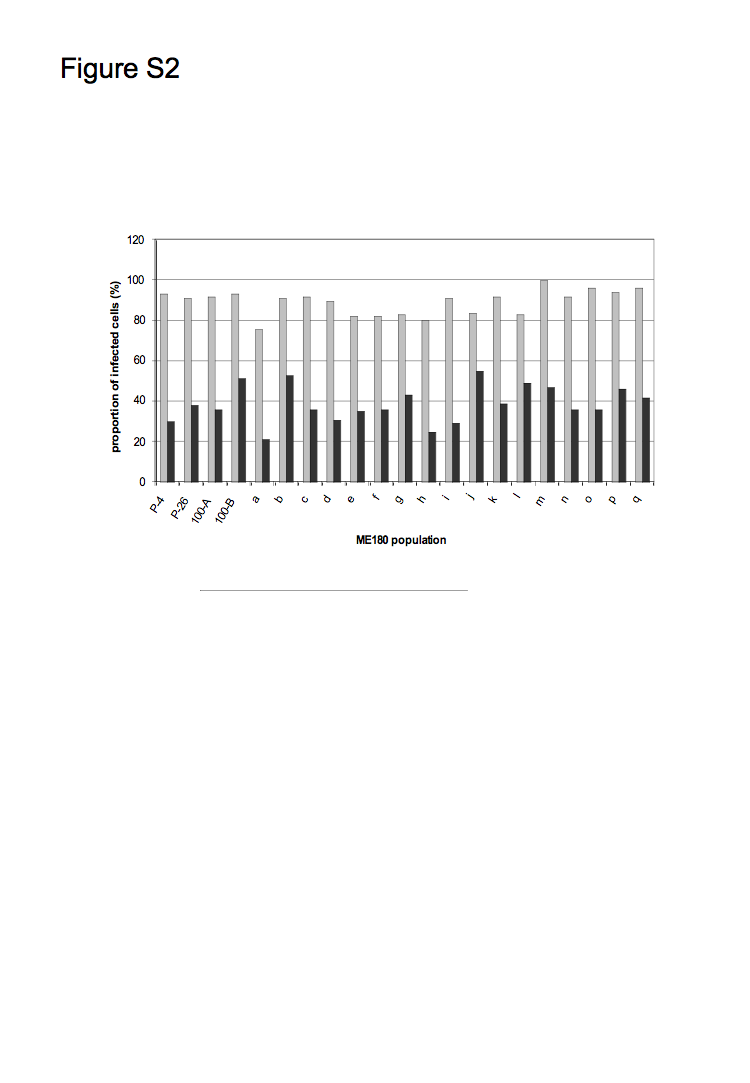

Supplement: Figure S2 — Clonal permissivity of ME180 cells to PilC1- or PilC2-mediated infection. Adhesion assays with either PilC1- or PilC2-expressing meningococci (strains Nm604a and 910f, respectively) were performed on 17 (a–q) single-cell derived ME180 populations (MOI 100). For each cellular clone, the percentage of cells permissive to infection is indicated for each meningococcal strain. Controls are early (p-4) and late (p-26) passages of ME180 cells, as well as two populations derived from 100 pooled ME180 single cells (100-A and 100-B). No difference was observed for single-cell derived populations and controls. (0.10 MB TIF) [file pone.0006834.s002.tif]
